# From Our Correspondents

**Published:** 1987-05

**Authors:** 


### From Our Correspondents

### Bristol Media Fund Cancer Research in Department of Surgery

During the last four months of 1986 the University Department of Surgery raised £100,000 by public appeal for cancer research. The particular project arose from a chance observation by Mr Nagy Habib and his colleagues at the Hammersmith Hospital that various malignant cells have a higher content of unsaturated fat than their normal counterparts. In particular, there is a relative increase in oleic acid (unsaturated) over stearic acid (saturated). This desaturation may confer an increased membrane fluidity on the tumour cell, in turn promoting cell metabolism and a greater mitotic rate. It had further been found in patients with different types of cancer that circulating red blood cells showed a similarly low saturation index (ratio of stearic to oleic acid), which tended to revert towards normal following complete surgical resection of the tumour. Thus the cancer appeared to be secreting a desaturation producing factor.

Sometime after the publication of these preliminary results a national Sunday newspaper reported the Hammersmith work in a somewhat sensational manner, suggesting extravagant claims for stearic acid therapy despite attempts by the surgical scientists concerned to moderate the article. The University Department of Surgery at the Bristol Royal Infirmary was linked with the Hammersmith Hospital in the newspaper report because by now Mr Habib had been appointed Lecturer in Surgery here. Although our Department has had continued funding from the Cancer Research Campaign for several years to support other projects, this particular grant application from the Hammersmith Hospital had been turned down. When the Bristol Evening Post and Radio Bristol learned (in the wake of the national publicity) that the project was under threat, they spontaneously launched an appeal for temporary funding to prevent it foundering. For whatever reason the appeal caught the public eye and the sum raised exceeded our expectations by a factor of 3 or 4. We have found this a moving testimony of the concern and perhaps the affection of the community for its local hospital and university.

As a direct consequence of the appeal monies, a surgical research fellow (Mr S Kelly) has been appointed to start work on the project in February 1987. In addition a technician can now be employed, a gas liquid chromatograph has been ordered (for the fatty acid analysis) and supplies of the expensive iodostearic acid can be secured. Clearly much work remains to be undertaken to pursue the basic biochemical defect in the tumour cell membrane. At the same time we have begun cautiously to explore the role of stearic acid supplements in restoring the saturation index to normal and inhibiting tumour growth. We do now have evidence that certain tumour cell lines can be inhibited in vitro and that carcinogenesis is partly blocked in an experimental model. With appropriate ethical approval iodostearic acid (iodination facilitates solubility) has been given to a very small number of patients with advanced cancer and without any ill effect. It is too soon to say if there has been any benefit. It is a tall order for any new therapy to make an impact on an established carcinoma towards the end of its growth curve. Among other things we have very little idea about optimal dose or route of administration.

At present I can only guess at the potential consequences of Nagy Habib's discovery. Perhaps toxicity or ineffectiveness will limit the application of fatty acid therapy in clinical practice, but at the very least we should learn more about the biology of the tumour cell and such information itself may lead to therapeutic advances. In the meantime though we did not seek publicity we have had a lucky break thanks to the generosity of Bristolians and others in the South West. It is now up to us to use the appeal money to the best possible end.

Robin Williamson

### The trials of trials

Advances in the practice of medicine and surgery are seldom made these days by chance observations or by using novel drugs or operations. Potential advances have to be considered carefully, planned with colleagues, funded by a local or national body, or a pharmaceutical firm, submitted to statisticians for their criticism, approved by a local ethical committee and validated by a controlled trial. Frequently a pilot study is needed in order to test feasibility and appropriate data recording. To acquire significant numbers usually means a multi-centre study with all the problems of paper work, parallel standards of care, and frequent meetings. To convince increasingly critical editors and readers, the trial usually has to be randomised and double blind.

There must be a prime mover of any trial, who needs strong motivation and determination: he or she must recruit as collaborators people who are like-minded and at least start as friends! He must consider most carefully how well his patients will co-operate in the study, particularly if this involves many extra visits to a clinic, venepunctures or consumption of unknown tablets! The chief investigator needs to enlist the co-operation of the nursing staff who may have to make additional observations on their patients, the hospital administration who may be involved in extra expenses, the junior medical staff and secretary who may have extra work imposed upon them.

Some of the problems which I have encountered in my career are:-

(1) The collaborator who wants to collect a lot of unnecessary data "just in case" - this usually results in an unmanageable protocol and recording system.

(2) The colleague who wants to change entry or exclusion criteria half way through the trial, which results in very woolly data.

(3) The co-trialist who loses interest half way through, and has to be chased repeatedly for patient details and results.

(4) The colleague who does not adhere to the agreed protocol or breaks the random code thus making many of the cases useless for analysis by introducing observer bias.

(5) The trial centre which declines to do further tests or examinations without substantial financial reward - this can scupper a study whether funded by a company or a research charity. It was a delight at a recent international planning committee to hear the "no problem" response of North American workers in contrast to the "well we can't do it without extra money" reply of some Europeans.

The reader may wonder why we embark on clinical trials, having read these comments. It is because I have a strong conviction that properly conducted trials are the only way we can advance many aspects of our practice. The history of medicine and surgery is littered with abandoned drugs, treatments and operations - recent examples being glucose potassium and insulin treatment for myocardial infarction, gastric freezing for bleeding ulcers and external-internal carotid anastomoses for cerebral ischaemia. Several of the trials in which I have been involved have proved to be negative, but they clear the air and prevent other teams wasting their time. One of the greatest problems is the escalating cost of proper trials, and I would like to pay tribute to the reputable companies who finance them, knowing that the results could be entirely unrewarding for them.

H. G. Mather

**MRI Scanner at Frenchay. Remarkable progress. Funds still needed.**

Further to the column in the December issue of the Bristol Medico-Chirurgical Journal from my colleague and fellow Trustee Paul Goddard about the M.R.I. Scanner, additional events have taken place since then which should be recorded.

The building is now completed and has been officially handed over by Cowlins to Picker International. Expert opinion has expressed considerable praise for the quality of the workmanship and the speed of construction carried out by Cowlins, and the Trustees would like to record their sincere thanks to all concerned. We would also like to thank Mr. Fred Dane, senior member of the Works Department at Frenchay Hospital and his assistant Mr. Graham Thomas, who have generously kept an eye on the developments over the months on behalf of the Trustees in an area completely beyond our scope and experience. The ground around the building has now also been landscaped and the approach road tarmaced, so that the scene has now become most attractive and full of yet further promise.

Readers will also like to know that we have appointed two radiographers to run the Scanner itself. Miss Ann Case will be in charge, and with over three years experience of working in the M.R.I. Research Centre at the Hammersmith Hospital, we anticipate that she will bring with her considerable knowledge of the fundamentals and applications of what to most of us appears to be a very difficult technology. We extend to her a warm welcome. Through her too, we hope to maintain a valuable link with the Hammersmith team and also the National Heart Hospital M.R.I. Centre, amongst others. Her support radiographer is Miss Joanne Waring from Addenbrooke's Hospital, Cambridge, who has done a great deal of work with CT, which provides the necessary experience with computers, and who has recently been on an M.R.I. Course run by the St. George's group at the Ciba Foundation in London. With these two permanent radiographers we hope to train at least three of our local Bristol radiographers over the course of the next year, so that we can contemplate the necessary staffing for out of hours and emergency cover when required, as it surely will be. In addition to these two radiographers we have appointed two part-time secretaries/receptionists, Mrs Gloria Hatton and Mrs Rona Wright both of whom already have considerable secretarial experience, but not yet in an M.R.I. Centre! All of these staff members will commence duties during the month of March, whilst the Scanner itself is being installed. We anticipate that the Centre will start to accept patients in the middle of April. Mr. John James generously gave the scanner to the Bristol Hospitals at the end of November 1985, so that the whole operation will have taken just over sixteen months. We hope he will be pleased with the venture when he sees the finished article. We ourselves feel that Bristol will be proud of it, and grateful to him for his gift.

From the financial aspect an added word may be of interest. The Chairman of the Trustees, Dr. Terry Beddoe, reported that the donations into the Fund have kept pace with the cost of the building, as indicated by the monthly bill from Cowlins. This is spendid news, and our grateful thanks are owed to so many people for their wonderful support. Over and above the cost of building, the whole of which has fallen to the Bristol M.R.I. Scanner Fund, we anticipate that the first year's running costs will be about £90,000, the full burden of which again falls on the Trustees to provide, so we desperately need continued support. In the second year of running the machine, during which time we then have to cover the Service Charge as an extra, our contribution from the Fund will need to be even more. However the N.H.S. has agreed to contribute 25% of the cost in year two, and on an increasing percentage basis thereafter, so that by year five, they will take over the complete running of the Centre. These financial facts must be borne in mind so that the general public and the medical profession can understand the implications of the continued Appeal for the Bristol M.R.I. Scanner Fund. And our grateful thanks extend to all those who can help.

Gordon Thomson

**Developments at St. Peter's Hospice**

St. Peter's Hospice "Sharing the Caring" is now an established part of the medical and nursing service of the County of Avon. The demand for its particular type of patient care has increased year by year since its inception in early 1978.

New beds and active Day Care Centre have been added during the last three years. First a Day Care Centre was established largely thanks to the generosity of the Anchor Society, and during the last two years a new building has been completed and is now in use, doubling the number of beds to fourteen, and allowing the Day Centre to move to larger accommodation in the old in-patient unit with greatly improved facilities. It is open four days a week, the patients being transported to and from their homes by volunteer drivers. The Day Centre releases Home Care nurses' time since people attending there do not require so many home visits yet they are regularly seen by medical and nursing staff. Domiciliary care is still a first priority in St. Peter's Hospice plan, and the new facilities support the Home Care service. The architect who designed the new building is a member of the Hospice Council and he and the builders used their skill and imagination to produce a homely building which, although modern, has a comfortable and comforting atmosphere in which patients are able to relax and the staff able to practice their special skills in sympton control, liberally salted with tender loving care. Space is now available for an extended educational programme, and the Hospice is now an established centre for the English Nursing Board Course 93 "Care of the dying patient and his family" as well as arranging Study Days for members of the caring professions, and students on such subjects as Sympton Control and Bereavement.

In March 1985 the Hospice was visited by the Duchess of Kent when she laid the foundation stone for the new building which she officially opened on 24th October 1986. The Duchess has graciously consented to be the Patron of St. Peter's and she shows very sincere and genuine concern for the welfare of those who benefit from Hospice care. The generosity of the people of Bristol and Avon give us all confidence for the future of the whole enterprise.

H. K. Bourns

**Burden Research Medal and Prize**

At the meeting on 10th June 1969 the Burden Trust instituted an annual award. "The Burden Research Medal and Prize", as a contribution to the Diamond Jubilee of the foundation of Stoke Park Hospital on 1st April, 1909, by the Rev, H. N. Burden and to encourage future research in the field of mental handicap. The Award consists of a gold medal and a prize of £500.

The Burden Research Medal and Prize is now being awarded biennially for outstanding research work which has been either published, accepted for publication, or presented as a paper to a learned society, during the proceeding three calendar years. The award is open to all registered medical practitioners, the greater part of whose time is spent working in the field of mental handicap in the United Kingdom or the Republic of Ireland.

The Awards Committee of the Burden Research Medal and Prize consists of the following members:–

(i) A member of the Burden Trust who is Chairman of the Awards Committee.
(ii) The Consultant Phsychiatrist in Charge of Stoke Park Hospital, who acts as the Secretary of the Awards Committee.
(iii) A representative of the Royal College of Psychiatrists who is the Chairman of the Section for the Psychiatry of Mental Handicap or a suitable person nominated by him.
(iv) A representative of Bristol University.
(v) A nominee of the Medical Research Council.

Winners of the Burden Research Medal and Prize, 1970 Dr B W Richards, 1971 Dr J Jancar, 1972 Dr G McCoull OBE, 1973 Dr P Sylvester, 1974 Dr V Cowie, 1975 Dr D A Primrose, 1978 Dr W A Heaton-Ward, 1980 Dr A H Reid, 1982 Dr J Corbett, 1984 Not Awarded, 1986 Dr K Day.

J. Jancar

**Writing**

When young writing often seemed a pain. Interminable essays with deadlines were for ever around. Just make a précis of Chapter 23 of H A L Fisher's *History of Europe* by half past three this afternoon. Later there was the matter of the 'paper chase' in medical journals. Something had got to get past the peer review system in order to achieve respectability. At first there is the temptation to be fashionable. Nowadays this means writing about monoclonal antibodies and genetic probes, subjects to which many senior authors of papers seem singularly ill-fitted.

Later on, with reviews and refereeing the same still holds good. There are the same deadlines, the same pain, and the need to bear in mind one's peers.

Does it, writing, become any easier? Well yes, I suppose so. But at what cost?

There are certain exceedingly boring tasks in writing which are infrequently admitted to. For myself, the prime bores are Minutes of Meetings and Grant Applications. I hope that no reader of this *Journal* will be too shocked to know that many contributions of mine to this column were written as light relief to those other, and awful stilted drafts. The language is different. At least I have never been guilty of calling anything 'customer-sensitive, bottom up', which I gather from a lunchtime companion is the fashionable way to talk about clinical services these days. Similar tortuous and poly-hyphenated ghouls have long inhabited the pages of Gowers' *Plain Words*: see the 2nd Complete Edition published 14 years ago before ACRONYMS really got going. Let the worst of these horrors rot quietly.

If anyone wants to know this piece is written instead of a Grant Application for yet another secretary to prop up the collapse of the University and the National Health Service. On reflection I think it would have made more bureaucratic sense to have put in for a pencil. At least you don't need to worry about whether it will get the expected support next year. Yes, writing can still be a pain, but why make it expensive as well?

J. D. Davies

**Machiavelli would have taken the bus**

One of the most important geographical advantages given to the Bristol Royal Infirmary, if in fact the only, is the close proximity of the central bus station. Each morning buses from every corner of Bristol and the surrounding countryside converge to the B.R.I.; since the introduction of the new, faster 'city dart' service these buses travel with increasing frequency and reliability. Whilst this magnificent public service makes us the envy of many other large teaching hospitals, how many of us doctors actually use it?

In his notorious book 'The Prince', Machiavelli outlined the foundations of 'shady' manoeuvring which can sometimes be witnessed in modern day politics. By his scheming and artful ploys he was able to keep one step ahead of his contemporaries in the conspiratorial Italy of the Renaissance. A born survivor, his skill was in seeing advantage where no-one else could. In reviewing the means of urban transport available, I have no doubt in speculating that if Machiavelli was alive today as a hospital doctor in Bristol, he would take the bus to work each morning and keep his car locked up in a garage. I present my evidence: (code S for senior doctors; J for juniors).

1) J. S.: As you safely reach work on time, you can take comfort knowing that some colleagues will be aimlessly floating up and down St Michael's Hill in their cars, searching for non-existent parking spaces and creating a public hazard.

2) J: "Sorry I'm late for the ward round sir, but the damned bus didn't turn up on time."

3) S: Bus fares are a quick and effective means of getting rid of small change.

4) J: When you board the city dart and the first twinges of insecurity appear as you anticipate the impending day's work, you will be able to adopt the mandatory foetal position without causing undue attention.

5) S: If your staff know that you get into work each morning by bus, will they really expect you to fund the 'Christmas outing'?

6) J: As the clinic grinds on past 8 p.m., you will be able to say to your boss with full sincerity and reluctance "I would love to stay and see some more patients, but the last bus home is shortly leaving."

7) S: "I'm sorry, darling, I would naturally love to nip into town and get the week's shopping, but I'm afraid that there just isn't any room on these new buses for heavy bags."

8) J: Attempting to read the morning tabloid within the small space allocated to your seat is excellent practice in surgical dexterity (reading the larger newspaper will require an assistant).

9) J. S.: Impeccable manners still exist in the twilight world of bus transport. Standing aside at bus queues, helping the elderly off and offering your seat up to a lady can all be witnessed. This is in stark contrast to the average rush hour car driver, laying eternal curses on their brethren, using Churchillian gestures as a form of recognition and playing various tunes on their car horn.

John McFadden

